# Anxiety disorder, depression and coronary artery disease: associations and modification by genetic susceptibility

**DOI:** 10.1186/s12916-025-03915-4

**Published:** 2025-02-06

**Authors:** Shinya Nakada, Joey Ward, Rona J. Strawbridge, Paul Welsh, Carlos Celis-Morales, Frederick K. Ho, Jill P. Pell

**Affiliations:** 1 School of Health and Wellbeing, University of Glasgow, 90 Byres Road, Glasgow, G12 8TB UK; 2 Division of Cardiovascular Medicine, Department of Medicine Solna, Karolinska Institute, Solna, Sweden; 3 HDR-UK, London, UK; 4 School of Cardiovascular and Metabolic Health, University of Glasgow, Glasgow, G12 8TA UK; 5 Human Performance Laboratory, Physical Activity and Health Research Unit, Universidad Católica del Maule, EducationTalca, Chile; 6 Centro de Investigación en Medicina de Altura (CEIMA), Universidad Arturo Prat, Iquique, Chile

**Keywords:** Depression, Anxiety, Coronary artery disease

## Abstract

**Background:**

Associations of anxiety disorder and depression with coronary artery disease (CAD) are heterogeneous between populations. This study investigated how genetic susceptibility to CAD alters these associations with incident CAD, comparing and combining anxiety disorder and depression.

**Methods:**

This is a prospective cohort study using UK Biobank. Diagnoses of anxiety disorder and depression were ascertained through linked hospital admission data. Incident CAD was ascertained through hospital admission and death certificate data after baseline. CAD polygenic risk score (PRS_CAD_) was obtained from CARDIoGRAMplus4 and categorised into low, intermediate, and high. Cox proportional hazard models were used to examine associations between anxiety disorder and depression and CAD.

**Results:**

Both anxiety disorder (HR 2.31, 95% CI 1.92–2.78) and depression (HR 2.15, 95% CI 1.90–2.24) were associated with CAD after adjusting for sociodemographic confounders. There was an addictive interaction between depression and PRS_CAD_ (RERI 0.97, 95% CI 0.12–1.81) such that the association between depression and CAD was strongest among those with a high PRS_CAD_ whilst there was no such evidence for anxiety disorder. Anxiety disorder only (HR 1.68, 95% 1.16–2.44), depression only (HR 2.13, 95% CI 1.72–2.64), and concomitant anxiety disorder and depression (HR 3.85, 95% CI 2.48–5.98) were associated with CAD even among people with a low PRS_CAD_. Adjusting for potential mediators attenuated all these associations across PRS categories.

**Conclusions:**

CAD genetic susceptibility might partly contribute to the clustering of depression and CAD but does not provide a full explanation, nor does it explain the association between anxiety disorder and CAD. Therefore, other mechanisms should be explored.

**Supplementary Information:**

The online version contains supplementary material available at 10.1186/s12916-025-03915-4.

## Background

Anxiety disorder and depression are the most common mental health conditions and substantial disease burdens globally [1]. Comorbid anxiety disorder and/or depression with coronary artery disease (CAD) is common and associated with higher mortality [2]. Previous studies reported that around one-third of patients with CAD suffered from anxiety disorder or depression [3] and they experienced two-fold higher mortality than people without these conditions [4]. One explanation for this comorbidity is that both anxiety disorder and depression increase the risk of CAD via pathways such as obesity and hypertension, as supported by accumulating studies [5–7].

However, the risk estimates vary between different populations. For example, the largest UK study found a moderate association between diagnosed depression and incident CAD (hazard ratio [HR] 1.52, 95% confidence interval [CI] 1.34–1.73) after adjusting for sociodemographic factors [8] whilst a Taiwanese study showed no association (HR 1.03, 95% CI 0.93–1.15) after similar adjustments [9]. These heterogeneous findings may be due to effect modifiers such as lifestyle or genetic factors that vary between populations. Previous studies have demonstrated that this association was modified by various lifestyle factors such as sleep duration [10–12]. CAD genetic variants could be associated with depression [13], suggesting that pleiotropy could account for the clustering of CAD and these mental health conditions [14]. If so, the likelihood of people with one condition also developing the second could be stronger in the presence of genetic predisposition.

A previous cohort study found that the association of the psychological well-being score with incident CAD were consistent across CAD polygenic risk score (PRS_CAD_) categories [15]. Another cohort study found an interaction between self-reported depressive mood and PRS_CAD_ for incident CAD [16]. There has been one cohort study into whether the association between depression and CAD was modified by PRS_CAD_ [17]. This study, based on 19,999 Finish participants, found no evidence of interaction. However, this could be due, in part, to being underpowered given its sample size. Furthermore, the focus of this study was on depression without considering anxiety disorder. Findings based on depression may not be fully applied to anxiety disorder. In addition, because these two mental health conditions can coexist, their joint conditions should be explored.

To this end, our study investigated the associations of anxiety disorder and/or depression with incident CAD overall and stratified by PRS_CAD_ in the UK Biobank cohort study.

## Methods

### Study design

This is a prospective cohort study using the data from UK Biobank which recruited over 500,000 UK residents aged 40 to 69 years from 2007 to 2010. Participants visited one of the 22 assessment centres across England, Scotland, and Wales to provide their information and undergo a series of examinations [18, 19]. We excluded participants who reported a history of cardiovascular diseases before the baseline assessment to reduce reverse causation, who were first admitted for anxiety disorder or depression after the baseline assessment, or who had missing data on sociodemographic confounders. We included only participants who self-reported their ethnicity as white.

### Measurements

Anxiety disorder and depression diagnosed before the baseline assessment were ascertained through record linkage to hospital admission data: Health Episode Statistics (England and Wales) and Scottish Morbidity Records (Scotland). We defined anxiety disorder as F40–43 and depression as F32–33, using the International Classification of Diseases, 10th revision (ICD-10).

Incident CAD events after the baseline assessment were ascertained through linkage to the hospital admission data and death certificate data: the National Health Service Information Centre (England and Wales) and the National Health Service Central Register Scotland (Scotland). The hospital admission data were available up to October 2022 in England, August 2022 in Scotland, and May 2022 in Wales and the death certificate data were available up to November 2022 in England, Wales, and Scotland. Follow-up was censored at the date of relevant hospitalisation or date of death, whichever occurred first. We defined CAD as an ICD-10 code of I20–25.

Sociodemographic confounders included age at recruitment, sex, educational level, and area deprivation index. Age, sex, and educational level were self-reported by participants using a touchscreen questionnaire at baseline. Educational level was dichotomised as college/university degree or any other. Area deprivation index was measured using the Townsend area deprivation index, which was derived from the postcode of residence using aggregated data on unemployment, car and home ownership, and household overcrowding [20].

Potential mediators included smoking status, body mass index (BMI), metabolic equivalent minutes (METs), diet quality, hypertension, low-density lipoprotein (LDL) cholesterol, hyperglycaemia, and C-reactive protein. We assumed that these factors measured at baseline lie on the path between anxiety disorder or depression and CAD. Smoking status was self-reported as current, previous, or never using the touchscreen questionnaire. METs per week were derived from the validated International Physical Activity Questionnaire. Diet quality was based on the cumulative dietary risk factors score, which has been reported previously [21]. Participants were given 1 point for each of nine dietary recommendations met relating to processed meat, red meat, total fish, milk, spread type, cereal intake, salt added to food, water, and fruits and vegetables based on the touchscreen questionnaire at baseline. The overall score ranged from 0 (least healthy) to 9 (most healthy). BMI was calculated as weight(kg)/height(m)^2^; height was measured to the nearest centimetre, using a Seca 202 stadiometer, and body weight was measured to the nearest 0.1 kg, using a Tanita BC-418 body composition analyser by trained staff. Hypertension was defined as systolic blood pressure ≥ 140 mmHg or taking antihypertensive medication [22]. Hyperglycaemia was defined as ≥ 48 mmol/mol or taking diabetic medication [23]. Elevated LDL cholesterol was defined as ≥ 4.9 mmol/L or taking cholesterol-lowering medication [24]. Biomarker (LDL cholesterol, haemoglobin A1c, and C-reactive protein) measurements were performed at a central laboratory between 2014 and 2017 and details of these assay performances are available in the protocol [25].

PRS_CAD_ was based on summary-level genetic data from CARDIoGRAMplus4 [26, 27] which is independent of the UK Biobank cohort. Ambiguous single-nucleotide polymorphisms were excluded from our analysis. PRS was calculated by adding up the number of risk alleles for each individual participant, selected based on a clumping and thresholding approach [28], then stratifying into tertiles labelled as low, intermediate, and high genetic scores. For the UK Biobank participants, genotyping, imputation, and standard quality control procedures were conducted centrally by the UK Biobank team. Genotyping was performed using the UK Biobank Axiom Array or UK BiLEVE Axiom Array and imputation was performed using a reference panel of HRC in combination with UK10K. All genetic quality control and computation of principal components of ancestry were undertaken by the central UK Biobank team [29].

### Statistical analyses

Participants’ characteristics were summarised by anxiety disorder and depression and PRS_CAD_, respectively, using frequency (percentage) for categorical data and mean (standard deviation) or median (interquartile range) for continuous data. All associations were estimated by Cox proportional hazard models, expressed as HRs and 95% CIs. Proportional hazard assumptions were checked using statistical tests based on Schoenfeld residuals. The main analyses were conducted in two stages. Firstly, the associations of anxiety disorder and/or depression with CAD were estimated conditional on sociodemographic confounders (age, sex, deprivation index, and educational level) and PRS_CAD_, genotype array, and 10 principal components of ancestry in two models: separate models included anxiety disorder and depression without mutual adjustment and joint models included isolated anxiety disorder and depression and their joint condition. Secondly, the results of the first stage were stratified by low, intermediate, and high PRS_CAD_. Multiplicative and additive interactions were tested by PRS_CAD_ tertiles and by extracting low and high PRS_CAD_ to examine whether the risk was different at extreme ends of the PRS_CAD_ distributions. Additional analyses were conducted to investigate whether including potential mediators (smoking status, BMI, METs, diet quality, hypertension, elevated LDL cholesterol, hyperglycaemia, and C-reactive protein) attenuated the associations across PRS_CAD_ categories. Sensitivity analyses were conducted by excluding those with severe mental health conditions, including schizophrenia and bipolar disorder and widening the definition of anxiety disorder and depression by including self-report of these conditions as the hospital admission record may not well capture milder cases. All analyses were conducted using R (version 3.5.3) with packages survival (version 3.2–7) and interactionR (version 0.1.5).

## Results

**Fig. 1:**
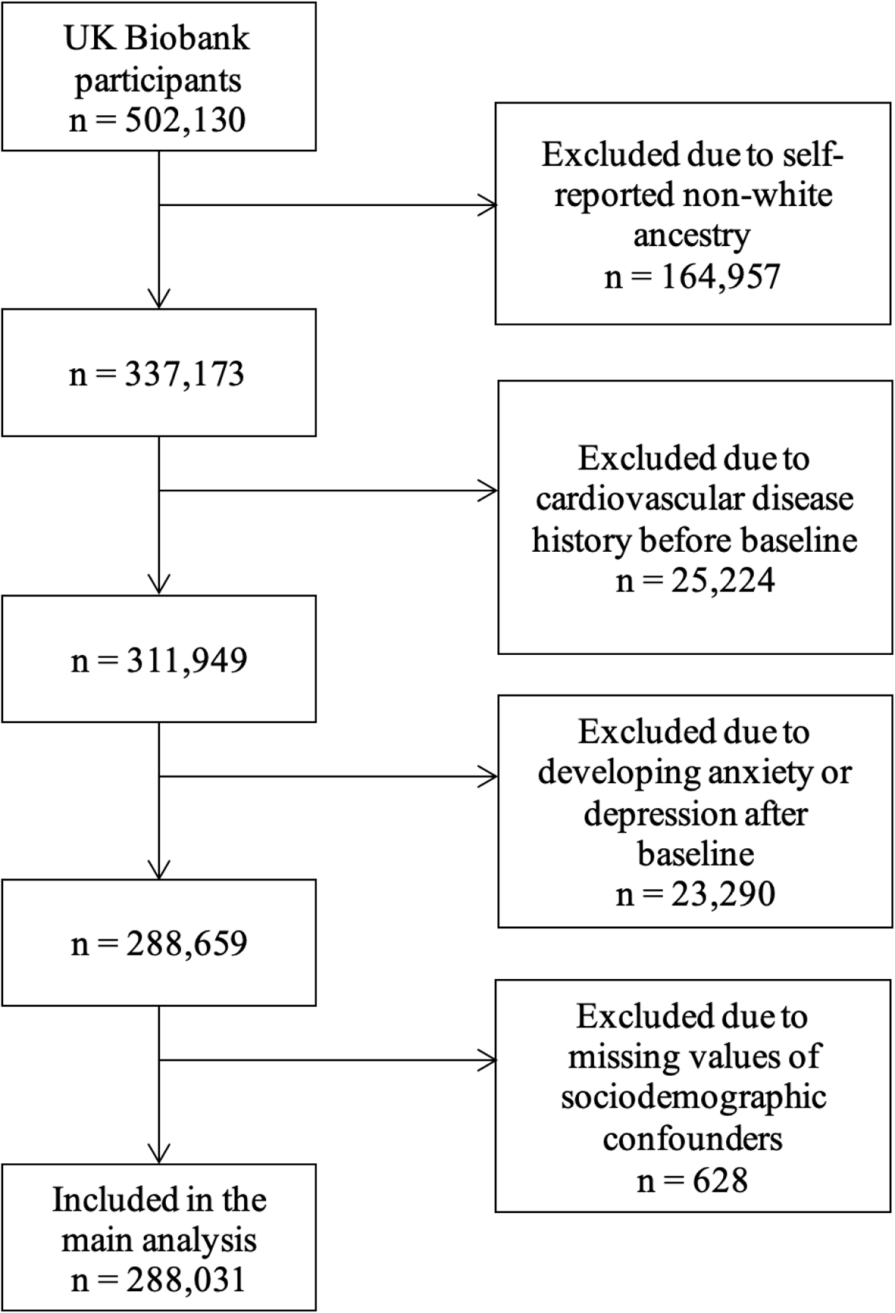
Flow chart of participant selection

Of the over 500,000 UK Biobank participants recruited, 288,031 were eligible for inclusion after excluding those 164,957 who self-reported an ethnicity other than white, 25,224 who had a history of cardiovascular disease, 23,290 who developed anxiety disorder or depression after the baseline assessment, and 628 who did not provide data on sociodemographic confounders (Fig. 1).

Overall, 779 (0.3%) had anxiety disorder and 1788 (0.6%) had depression; 557 (0.2%) had isolated anxiety disorder, 1566 (0.5%) had isolated depression, and 222 (0.1%) had both conditions. Participants with anxiety disorder or depression were more likely to be younger, female, more deprived and less likely to attain higher education, compared with those without these conditions (Table 1). They were also more likely to experience a CAD event (Supplementary Fig. 1, Supplementary Table 1); 112 (14.5%) of those with anxiety disorder and 21,212 (7.4%) of those without experienced an event over a median follow-up time of 13.4 and 13.6 years, respectively; 238 (13.4%) of those with depression and 21,086 (7.4%) of those without experienced an event over a median follow-up time of 13.3 and 13.6 years, respectively. Participants within the higher PRS_CAD_ categories were slightly more likely to be female and less likely to attain higher education (Supplementary Table 2) and they were more likely to experience a CAD event (Supplementary Fig. 1).

**Table 1:** Participant characteristics by anxiety disorder and depression

	**No. (%) of participants**^**a**^			
	**No anxiety disorder** *N* = 287,252	**Anxiety disorder** *N* = 779	**No depression** *N* = 286,243	**Depression** *N* = 1788
Age (years), mean (SD)	56.5 (7.98)	55.4 (8.17)	56.5 (7.98)	55.2 (7.88)
Sex				
Female	156,430 (54.46)	486 (62.39)	155,820 (54.44)	1096 (61.30)
Male	130,822 (45.54)	293 (37.61)	130,423 (45.56)	692 (38.70)
Deprivation index, mean (SD)	− 1.7 (2.87)	− 0.6 (3.34)	− 1.7 (2.86)	− 0.1 (3.56)
With college or University degree	95,463 (33.23)	181 (23.23)	95,194 (33.26)	450 (25.17)
Polygenic risk score, mean (SD)	3036 (47.46)	3039 (46.40)	3036 (47.45)	3038 (48.30)
Polygenic risk score				
Low	95,769 (33.34)	242 (31.07)	95,409 (33.33)	602 (33.67)
Intermediate	95,750 (33.33)	260 (33.38)	95,438 (33.34)	572 (31.99)
High	95,733 (33.33)	277 (35.56)	95,396 (33.33)	614 (34.34)
Smoking				
Never	161,610 (56.43)	423 (54.44)	161,234 (56.50)	799 (44.96)
Previous	97,713 (34.12)	216 (27.80)	97,360 (34.11)	569 (32.02)
Current	27,066 (9.45)	138 (17.76)	26,795 (9.39)	409 (23.02)
Missing	863	2	854	11
MET-minutes/week, median (IQR)	1848 (2825)	1864 (3034)	1850 (2825)	1518 (2794)
Missing	60,677	215	60,416	476
Diet quality score, mean (SD)	4.4 (1.60)	4.4 (1.67)	4.4 (1.60)	4.2 (1.68)
Missing	33,563	97	33,419	241
Body mass index (kg/m^2^), mean (SD)	27.2 (4.62)	27.7 (5.28)	27.2 (4.61)	28.8 (5.97)
Missing	778	7	771	14
SBP ≥ 140 mmHg or medication	139,469 (49.79)	366 (48.48)	138,989 (49.79)	846 (48.79)
Missing	7134	24	7104	54
LDL-c ≥ 4.9 mmol/L or medication	55,293 (20.08)	173 (23.35)	54,997 (20.04)	469 (27.33)
Missing	11,875	38	11,841	72
HbA1c ≥ 48 mmol/mol or medication	8086 (2.95)	32 (4.26)	8025 (2.94)	93 (5.44)
Missing	13,056	27	13,005	78
C-reactive protein (mg/L), median (IQR)	1.28 (2.0)	1.65 (2.65)	1.28 (2.0)	1.96 (3.22)
Missing	13,376	43	13,331	88

*SD* Standard deviation, *IQR* Interquartile range, *MET* Metabolic equivalent, *SBP* Systolic blood pressure, *HbA1c* Haemoglobin A1c, *LDL-c* Low-density lipoprotein cholesterol

^a^Unless indicated otherwise

In the univariable and multivariable models adjusted for sociodemographic confounders, both anxiety disorder and depression were associated with CAD with the strongest association for concomitant anxiety disorder and depression (Table 2). After additional adjustments for PRS_CAD_, the strengths of these associations were only slightly reduced.

**Table 2:** Associations between diagnosed anxiety disorder and depression and coronary artery disease

	Model 1	Model 2	Model 3
	HR (95% CI)	HR (95% CI)	HR (95% CI)
Separate models^a^ (*n* = 288,031)			
Anxiety disorder (± depression)	2.09 (1.74–2.52)	2.31 (1.92–2.78)	2.28 (1.89–2.74)
Depression (± anxiety disorder)	1.97 (1.73–2.24)	2.15 (1.90–2.45)	2.14 (1.88–2.43)
Combined model (*n* = 288,031)			
Anxiety disorder only	1.88 (1.50–2.36)	2.06 (1.64–2.59)	2.03 (1.61–2.55)
Depression only	1.87 (1.63–2.15)	2.04 (1.78–2.35)	2.03 (1.76–2.33)
Both anxiety disorder and depression	2.71 (1.98–3.70)	3.05 (2.23–4.17)	3.04 (2.22–4.16)

Model 1: no adjustment

Model 2: adjusted for age, sex, deprivation index, and education

Model 3: adjusted for age, sex, deprivation index, education, polygenic risk score, genotyping chip, and 10 principal components

*n*, number; *HR*, hazard ratio; *CI*, confidence interval

^a^Anxiety disorder and depression were not mutually adjusted

**Fig. 2:**
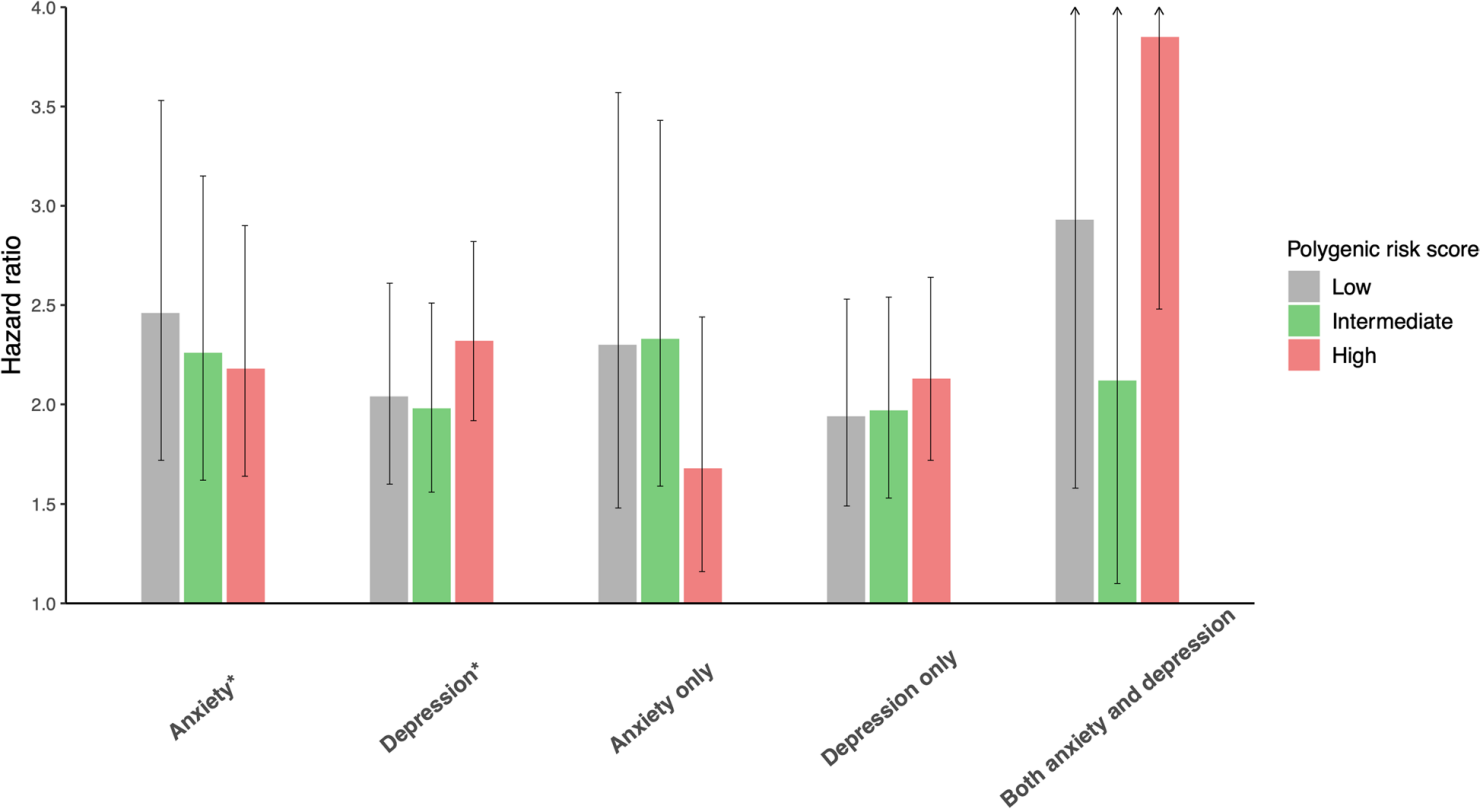
Associations between diagnosed anxiety disorder and depression and coronary artery disease by polygenic risk score. *Anxiety disorder and depression were not mutually adjusted. All models were adjusted for age, sex, deprivation index, education, genotyping chip, and 10 principal components

After stratifying by PRS_CAD_, anxiety disorder was associated with CAD across low, intermediate, and high PRS_CAD_ categories with the strongest associations in the low PRS_CAD_ category (Table 3 and Fig. 2). In contrast, the association between depression and CAD was strongest among those in the high PRS_CAD_ category with evidence of additive interaction (RERI 0.97, 95% CI 0.12–1.81). The associations between concomitant anxiety disorder and depression and CAD were strong in both the high PRS_CAD_ category (HR 3.85, 95% CI 2.48–5.98) and low PRS_CAD_ category (HR 2.93, 95% CI 1.58–5.46). Repeating the analysis after excluding those with prevalent severe mental health conditions yielded consistent results (Supplementary Table 3). Additional adjustments for potential mediators attenuated the magnitude of the associations in both the separate and joint models and across PRS_CAD_ categories but the associations remained (Supplementary Table 4).

**Table 3:** Associations between diagnosed anxiety disorder and depression and coronary artery disease by polygenic risk score

	Low PRS	Intermediate PRS	High PRS	Multiplicative interaction	Additive interaction
	HR (95% CI)	HR (95% CI)	HR (95% CI)	HR (95% CI)	RERI (95% CI)
Separate models^a^ (*n* = 288,031)					
Anxiety disorder (± depression)	2.46 (1.72–3.53)	2.26 (1.62–3.15)	2.18 (1.64–2.90)	0.87 (0.55–1.38)	0.31 (− 0.99–1.60)
Depression (± anxiety disorder)	2.04 (1.60–2.61)	1.98 (1.56–2.51)	2.32 (1.92–2.82)	1.13 (0.83–1.54)	0.97 (0.12–1.81)
Combined model (*n* = 288,031)					
Anxiety disorder only	2.30 (1.48–3.57)	2.33 (1.59–3.43)	1.68 (1.16–2.44)	0.71 (0.40–1.27)	− 0.31 (− 1.71–1.08)
Depression only	1.94 (1.49–2.53)	1.97 (1.53–2.54)	2.13 (1.72–2.64)	1.08 (0.77–1.52)	0.76 (− 0.10–1.63)
Both anxiety disorder and depression	2.93 (1.58–5.46)	2.12 (1.10–4.07)	3.85 (2.48–5.98)	1.33 (0.62–2.84)	2.48 (− 0.68–5.65)

All models were adjusted for age, sex, deprivation index, education, genotyping chip, and 10 principal components

Interaction was calculated from low and high PRS categories

*n* number, *HR* Hazard ratio, *CI* Confidence interval, *PRS* Polygenic risk score, *RERI* Relative excess risk due to interaction

^a^Anxiety disorder and depression were not mutually adjusted

Expanding ascertainment of anxiety disorder and depression to include self-reported disease attenuated their associations with CAD and adjusting for PRS_CAD_ did not change the estimates (Supplementary Table 5). In both separate and joint models, as with anxiety disorder, the estimated effect sizes for depression varied little by PRS_CAD_ category (Supplementary Table 6). Overall, additional adjustments for potential mediators slightly attenuated the magnitude of associations across PRS_CAD_ categories (Supplementary Table 7).

## Discussion

### Primary findings

Our findings demonstrated that the association between depression and CAD was stronger among those with a high PRS_CAD_. In contrast, this pattern was not evident for anxiety disorder nor concomitant anxiety disorder and depression which were associated with CAD regardless of genetic predisposition to CAD. Adjusting for potential mediators attenuated these associations between anxiety disorder and/or depression and CAD, across PRS_CAD_ categories, consistent with them acting as mediators.

### Comparisons with previous studies

We investigated the associations of anxiety disorder and depression with incident CAD and their modification by PRS_CAD_ in a single, very large, prospective, general population cohort study. We ascertained these mental health conditions through record linkage to hospital admission data as ICD-10 codes to obviate any reporting or recall bias. We also established the temporal order of these mental health conditions, potential mediators, and CAD to obviate reverse causation. A possible interaction between depression and PRS_CAD_ has been investigated in only one existing study [17]. The non-significant finding of that study differ from ours and may be due to being underpowered by a much smaller sample size and testing for a multiplicative interaction alone. Furthermore, the authors acknowledged their potential measurement errors of depression due to the reliance on purchasing records of antidepressants, which is not specific to depression because of their broad indications.

Our findings offer several interpretations. Firstly, the stronger association between depression and CAD among those with genetic susceptibility would be consistent with pleiotropy contributing to the clustering of these conditions as shown by previous studies reporting the shared genetic architecture [13, 14, 30, 31]. For example, a recent UK study found a strong genetic correlation between depression and CAD (r_g_ = 0.56) and similarly diabetes, hypertension, BMI, and waist-hip ratio [30]. However, this did not fully explain the observed association between depression and CAD in those with low genetic predisposition, nor does it explain the associations between anxiety disorder and CAD, implying other mechanisms must play a role in the clustering of these mental health conditions and CAD. Adjustment for potential mediators attenuated the associations between these mental health conditions and CAD, consistent with previous studies [32–34]. These findings are consistent with mediation contributing to the clustering, whereby these mental health conditions adversely impact lifestyle which, in turn, predisposes to CAD. This is supported by previous evidence, such that people with depression and anxiety disorder are more likely to smoke [35].

Secondly, anxiety disorder and depression may respond to CAD genetic predisposition differently. This may be surprising given their genetic and phenotypic similarities. Anxiety disorder and depression have a strong genetic correlation (r_g_ = 0.90) and have sometimes been classified in the same psychiatric group as internalising disorders [36, 37]. In addition, they often co-exist and share similar symptoms. Around 40% of people with depression experienced anxiety disorder during 12-month follow-up and 45% over their lifetime [38]. They shared many symptoms such as low energy and disturbed appetite, which could support their similar association strengths with CAD in our study [39]. On the other hand, there seem distinct mechanisms by which each of these mental health conditions increases CAD risk [7]. For example, whilst high blood pressure is more specific to anxiety disorder [40], inflammation is more associated with depression (particularly in the immunometabolic subtype) [41], and this may result in our contrasting findings in relation to modification.

Thirdly, the role of depression in CAD development may be heterogeneous in that, overall, we found inconsistent results between diagnosed depression and additional use of self-reports. One reason is the varying severity between different measurements of depression: diagnosed depression in a hospital is probably more severe than self-reported depression which includes cases managed exclusively in the community. Interestingly, our study has suggested that the varying severity not only affects the size of the CAD risk but also affects the risk modification by CAD genetic susceptibility. This would encourage further studies to understand the underlying mechanisms and how to identify high-risk groups among those suffering from mental health disorders.

### Limitations

Whilst there were several strengths in our study, the following limitations should be observed when interpreting the results. Firstly, remitting–relapsing fluctuations of these mental health conditions may affect our estimates. Because we only ascertained whether anxiety disorder and depression had ever occurred before the baseline assessment, it is unclear whether or not they remitted over follow-up and if so, how this attenuated our estimates [42]. Secondly, our study based the diagnoses of these mental health conditions on the ICD-10 classification system, which cannot be directly mapped to the Diagnostic and Statistical Manual of Mental Disorders (DSM) classifications. Therefore, our findings may have limited applicability in settings that adopt the DSM system [43]. Thirdly, our main findings may not fully apply to milder cases of anxiety disorder and depression, as shown in the sensitivity analysis. Fourthly, because the original GWAS predominantly included those with European ancestry, our study was restricted to those reported as white. Therefore, our findings may not apply to other ethnic groups. Fifthly, residual confounding remains a concern due to unmeasured confounders as with other observational studies. Lastly, anxiety disorder and depression in this study were less prevalent than in previous general population studies [44], indicating that caution is required when applying the study findings outside UK Biobank. There are potentially two reasons for such discrepancies: (1) the UK Biobank participants do not fully represent the general population because they tend to be healthier [45], and (2) hospital admission data were used to ascertain these mental health conditions, which are mainly the more severe cases.

### Implications

Because the prevalence of anxiety disorder, depression, and CAD are increasing [1, 46], understanding the mechanism behind their mental health-cardiovascular comorbidity is needed to reduce the public health burden. Our findings suggest that CAD genetic susceptibility contributes to the clustering of depression and CAD by modifying their associations but does not provide the full explanation. Clustering is likely to also be due, in part, to poor mental health being associated with unhealthy lifestyle choices and their downstream biomarkers which predispose to CAD. The latter mechanism suggests that this form of multimorbidity could potentially be tackled by modifying those mediating factors in addition to treating and preventing anxiety disorder and depression.

## Conclusions

CAD genetic susceptibility might partly contribute to the clustering of depression and CAD but does not provide a full explanation, nor does it explain the association between anxiety disorder and CAD. Therefore, other mechanisms should be explored.

## Supplementary Information

Supplementary Material 1.

## Data Availability

No datasets were generated or analysed during the current study.

## Abbreviations

CAD: Coronary artery disease
HR: Hazard ratio
CI: Confidence interval
PRS_CAD_: Coronary artery disease polygenic risk score
ICD-10: International Classification of Diseases, 10th revision
BMI: Body mass index
Mets: Metabolic equivalent minutes
LDL: Low-density lipoprotein
DSM: Diagnostic and Statistical Manual of Mental Disorders
