# Supplementary material for: Anxiety disorder, depression and coronary artery disease: associations and modification by genetic susceptibility
Source: BMC Med. 2025 Feb 6;23:73. doi: 10.1186/s12916-025-03915-4 (PMC11804096; doi:10.1186/s12916-025-03915-4)
Supplement: Supplementary file 1 — Supplementary Material 1. [file 12916_2025_3915_MOESM1_ESM.docx]

**Anxiety disorder, depression and coronary artery disease: associations and modification by genetic susceptibility**

Supplementary Figure 1. Kaplan-Meier estimates of the survivor function for coronary artery disease comparing participants by anxiety disorder, depression, and polygenic risk score

Supplementary Table 1. Number of coronary artery disease events during the study period

Supplementary Table 2. Participant characteristics by coronary artery disease polygenic risk score

Supplementary Table 3. Associations between diagnosed anxiety disorder and depression and coronary artery disease by polygenic risk score after excluding participants with severe mental health conditions

Supplementary Table 4. Associations between diagnosed anxiety disorder and depression and coronary artery disease by polygenic risk score, adjusted for potential mediators

Supplementary Table 5. Associations between diagnosed or self-reported anxiety disorder and depression and coronary artery disease

Supplementary Table 6. Associations between diagnosed or self-reported anxiety disorder and depression and coronary artery disease by polygenic risk score

Supplementary Table 7. Associations between diagnosed or self-reported anxiety disorder and depression and coronary artery disease by polygenic risk score, adjusted for potential mediators

Supplementary Figure 1. Kaplan-Meier estimates of the survivor function for coronary artery disease comparing participants by anxiety disorder, depression, and polygenic risk score


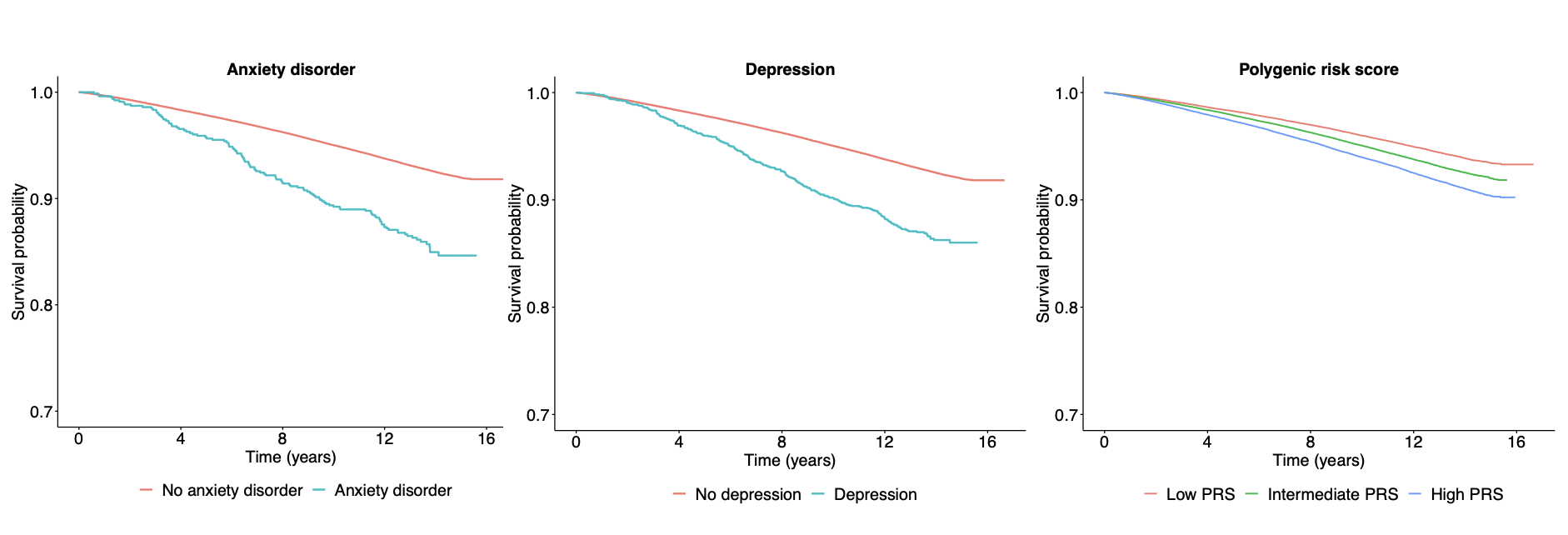


Supplementary Table 1. Number of coronary artery disease events during the study period

|  | 2 years | 4 years | 6 years | 8 years | 10 years | 12 years | 14 years | 16 years | Total |
| --- | --- | --- | --- | --- | --- | --- | --- | --- | --- |
| No anxiety disorder | 2,104 | 2,730 | 2,855 | 3,107 | 3,505 | 3,631 | 2,847 | 433 | 21,212 |
| Anxiety disorder | 9 | 18 | 13 | 26 | 18 | 15 | 13 | 0 | 112 |
| No depression | 2,096 | 2,710 | 2,834 | 3,091 | 3,478 | 3,612 | 2,832 | 433 | 21,086 |
| Depression | 17 | 38 | 34 | 42 | 45 | 34 | 28 | 0 | 238 |

Supplementary Table 2. Participant characteristics by coronary artery disease polygenic risk score

|  | Low PRS  N = 96,011^1^ | Intermediate PRS  N = 96,010^1^ | High PRS  N = 96,010^1^ |
| --- | --- | --- | --- |
| Age (years), mean (SD) | 56.6 (7.98) | 56.5 (7.98) | 56.4 (7.98) |
| Sex |  |  |  |
| Female | 51,766 (53.92) | 52,265 (54.44) | 52,884 (55.08) |
| Male | 44,245 (46.08) | 43,745 (45.56) | 43,126 (44.92) |
| Deprivation index, mean (SD) | -1.7 (2.86) | -1.7 (2.87) | -1.7 (2.87) |
| With college or University degree | 32,719 (34.08) | 31,793 (33.11) | 31,131 (32.43) |
| Smoking |  |  |  |
| Never | 54,245 (56.67) | 53,783 (56.18) | 54,006 (56.42) |
| Previous | 32,581 (34.04) | 32,783 (34.25) | 32,564 (34.02) |
| Current | 8,895 (9.29) | 9,164 (9.57) | 9,145 (9.55) |
| Missing | 290 | 280 | 295 |
| MET-minutes/week, median (IQR) | 1,830 (2,801) | 1,855 (2,828) | 1,860 (2,848) |
| Missing | 19,997 | 20,270 | 20,625 |
| Diet quality score, mean (SD) | 4.4 (1.60) | 4.4 (1.61) | 4.4 (1.61) |
| Missing | 11,422 | 11,123 | 11,115 |
| Body mass index (kg/m2), mean (SD) | 27.1 (4.58) | 27.2 (4.63) | 27.3 (4.66) |
| Missing | 254 | 254 | 277 |
| SBP ≥140mmHg or medication | 44,898 (47.97) | 46,719 (49.93) | 48,219 (51.46) |
| Missing | 2,409 | 2,433 | 2,316 |
| LDL-c ≥ 4.9 mmol/L or medication | 16,592 (18.03) | 18,518 (20.14) | 20,356 (22.10) |
| Missing | 3,962 | 4,061 | 3,890 |
| HbA1c ≥ 48 mmol/mol or medication | 2,500 (2.73) | 2,755 (3.01) | 2,863 (3.12) |
| Missing | 4,442 | 4,367 | 4,274 |
| C-reactive protein (mg/L), median (IQR) | 1.25 (1.97) | 1.28 (2.01) | 1.31 (2.04) |
| Missing | 4,401 | 4,567 | 4,451 |

^1^Numbers presented are number (%) unless otherwise stated; SD, standard deviation; IQR, interquartile range; PRS, polygenic risk score; MET, metabolic equivalent; SBP, systolic blood pressure; HbA1c, haemoglobin A1c; LDL-c, low-density lipoprotein cholesterol

Supplementary Table 3. Associations between diagnosed anxiety disorder and depression and coronary artery disease by polygenic risk score after excluding participants with severe mental health conditions

|  | Low PRS | Intermediate PRS | High PRS | Multiplicative  interaction | Additive interaction |
| --- | --- | --- | --- | --- | --- |
|  | HR (95% CI) | HR (95% CI) | HR (95% CI) | HR (95% CI) | RERI (95% CI) |
| Separate models* (n=287,608) |  |  |  |  |  |
| Anxiety disorder (+/- depression) | 2.50 (1.73–3.60) | 2.25 (1.61–3.15) | 2.11 (1.56–2.84) | 0.83 (0.52–1.33) | 0.15 (-1.18–1.48) |
| Depression (+/- anxiety disorder) | 2.01 (1.55–2.59) | 1.85 (1.44–2.39) | 2.23 (1.82–2.73) | 1.10 (0.79–1.52) | 0.84 (-0.01–1.70) |
| Combined model (n=287,608) |  |  |  |  |  |
| Anxiety disorder only | 2.35 (1.51–3.65) | 2.35 (1.60–3.45) | 1.67 (1.15–2.44) | 0.69 (0.39–1.24) | -0.39 (-1.81–1.04) |
| Depression only | 1.90 (1.44–2.51) | 1.84 (1.40–2.41) | 2.05 (1.65–2.57) | 1.06 (0.75–1.52) | 0.68 (-0.19–1.56) |
| Both anxiety disorder and depression | 2.97 (1.54–5.72) | 2.01 (1.01–4.03) | 3.87 (2.36–6.33) | 1.31 (0.58–2.98) | 2.44 (-1.04–5.92) |

n, number; HR, hazard ratio; CI, confidence interval; PRS, polygenic risk score; RERI: relative excess risk due to interaction

* Anxiety disorder and depression were not mutually adjusted.

All models were adjusted for age, sex, deprivation index, education, genotyping chip, and 10 principal components.

Interaction was calculated from Low and High PRS categories.

Supplementary Table 4. Associations between diagnosed anxiety disorder and depression and coronary artery disease by polygenic risk score, adjusted for potential mediators

|  | Low PRS | Intermediate PRS | High PRS |
| --- | --- | --- | --- |
|  | HR (95% CI) | HR (95% CI) | HR (95% CI) |
| Separate models* (n=179,555) |  |  |  |
| Anxiety disorder | 1.69 (0.98–2.91) | 2.10 (1.34–3.30) | 1.97 (1.36–2.86) |
| Depression | 1.68 (1.20–2.35) | 1.73 (1.25–2.40) | 1.75 (1.33–2.29) |
| Combined model (n=179,555) |  |  |  |
| Anxiety disorder only | 1.45 (0.72–2.91) | 1.91 (1.08–3.38) | 1.49 (0.91–2.44) |
| Depression only | 1.61 (1.12–2.31) | 1.61 (1.12–2.31) | 1.53 (1.13–2.08) |
| Both anxiety disorder and depression | 2.32 (0.96–5.58) | 2.56 (1.22–5.38) | 3.50 (1.98–6.19) |

n, number; HR, hazard ratio; CI, confidence interval; PRS, polygenic risk score

* Anxiety disorder and depression were not mutually adjusted.

Models were adjusted for age, sex, deprivation index, education, genotyping chip, 10 principal components, smoking status, MET, diet quality score, body mass index, hypertension, high low-density lipoprotein cholesterol, hyperglycaemia, and C-reactive protein.

Supplementary Table 5. Associations between diagnosed or self-reported anxiety disorder and depression and coronary artery disease

|  | Model 1 | Model 2 | Model 3 |
| --- | --- | --- | --- |
|  | HR (95% CI) | HR (95% CI) | HR (95% CI) |
| Separate models* (n=288,031) |  |  |  |
| Anxiety disorder (+/- depression) | 1.13 (1.02–1.26) | 1.25 (1.13–1.39) | 1.26 (1.13–1.39) |
| Depression (+/- anxiety disorder) | 1.02 (0.96–1.09) | 1.25 (1.17–1.34) | 1.25 (1.17–1.34) |
| Combined model (n=288,031) |  |  |  |
| Anxiety disorder only | 1.09 (0.97–1.23) | 1.20 (1.06–1.35) | 1.20 (1.06–1.35) |
| Depression only | 1.00 (0.93–1.07) | 1.23 (1.15–1.32) | 1.23 (1.15–1.32) |
| Both anxiety disorder and depression | 1.25 (1.03–1.52) | 1.47 (1.21–1.78) | 1.48 (1.22–1.79) |

n, number; HR, hazard ratio; CI, confidence interval

* Anxiety disorder and depression were not mutually adjusted.

Model 1: no adjustment.

Model 2: adjusted for age, sex, deprivation index, and education.

Model 3: adjusted for age, sex, deprivation index, education, polygenic risk score, genotyping chip, and 10 principal components.

Supplementary Table 6. Associations between diagnosed or self-reported anxiety disorder and depression and coronary artery disease by polygenic risk score

|  | Low PRS | Intermediate PRS | High PRS | Multiplicative  interaction | Additive interaction |
| --- | --- | --- | --- | --- | --- |
|  | HR (95% CI) | HR (95% CI) | HR (95% CI) | HR (95% CI) | RERI (95% CI) |
| Separate models* (n=288,031) |  |  |  |  |  |
| Anxiety disorder (+/- depression) | 1.29 (1.07–1.57) | 1.24 (1.03–1.48) | 1.25 (1.06–1.47) | 0.97 (0.75, 1.24) | 0.09 (-0.30, 0.49) |
| Depression (+/- anxiety disorder) | 1.27 (1.12–1.44) | 1.21 (1.08–1.36) | 1.27 (1.15–1.41) | 1 (0.85, 1.18) | 0.15 (-0.1, 0.41) |
| Combined model (n=288,031) |  |  |  |  |  |
| Anxiety disorder only | 1.29 (1.03–1.61) | 1.25 (1.01–1.53) | 1.10 (0.91–1.34) | 0.86 (0.63, 1.16) | -0.13 (-0.57, 0.32) |
| Depression only | 1.27 (1.11–1.45) | 1.21 (1.07–1.37) | 1.23 (1.10–1.37) | 0.97 (0.82, 1.15) | 0.08 (-0.18, 0.35) |
| Both anxiety disorder and depression | 1.36 (0.94–1.98) | 1.24 (0.86–1.77) | 1.80 (1.35–2.40) | 1.32 (0.83, 2.12) | 0.88 (-0.07, 1.82) |

n, number; HR, hazard ratio; CI, confidence interval; PRS, polygenic risk score

* Anxiety disorder and depression were not mutually adjusted.

All models were adjusted for age, sex, deprivation index, education, genotyping chip, and 10 principal components.

Supplementary Table 7. Associations between diagnosed or self-reported anxiety disorder and depression and coronary artery disease by polygenic risk score, adjusted for potential mediators

|  | Low PRS | Intermediate PRS | High PRS |
| --- | --- | --- | --- |
|  | HR (95% CI) | HR (95% CI) | HR (95% CI) |
| Separate models* (n=179,555) |  |  |  |
| Anxiety disorder | 1.28 (0.99–1.66) | 1.25 (0.99–1.58) | 1.22 (0.99–1.50) |
| Depression | 1.23 (1.05–1.45) | 1.07 (0.91–1.25) | 1.14 (0.99–1.31) |
| Crude model (n=179,555) |  |  |  |
| Anxiety disorder only | 1.25 (0.93–1.70) | 1.23 (0.94–1.62) | 1.10 (0.86–1.40) |
| Depression only | 1.22 (1.03–1.44) | 1.05 (0.89–1.24) | 1.09 (0.94–1.26) |
| Both anxiety disorder and depression | 1.41 (0.87–2.27) | 1.31 (0.83–2.05) | 1.74 (1.17–2.58) |

n, number; HR, hazard ratio; CI, confidence interval; PRS, polygenic risk score

* Anxiety disorder and depression were not mutually adjusted.

Models were adjusted for age, sex, deprivation index, education, genotyping chip, 10 principal components, smoking status, MET, diet quality score, body mass index, hypertension, high low-density lipoprotein cholesterol, hyperglycaemia, and C-reactive protein.
